# Determinants of trajectories of informal caregiving in later life: evidence from England

**DOI:** 10.1007/s10433-024-00818-w

**Published:** 2024-08-31

**Authors:** Giorgio Di Gessa, Christian Deindl

**Affiliations:** 1 Department of Epidemiology and Public Health, University College London, 1-19 Torrington Place, London, WC1E 7HB UK; 2 Department of Social Sciences, TU Dortmund University, Dortmund, Germany

**Keywords:** Provision of informal care, Caregiver, Care provision, Caregiving, Patterns, Trajectories, Longitudinal, Family composition, Partner’s health

## Abstract

Although the long-term consequences of informal care provision have been well investigated, few studies have examined the trajectories of informal care provision among older people and the socioeconomic, demographic, health, and family characteristics associated with them. We use data from four waves of the English Longitudinal Study of Ageing, with 6561 respondents followed for 6 years (2012/3–2018/9). We used group-based trajectory modelling to group people's provision of care over time into a finite number of distinct trajectories of caregiving. Using multinomial logistic regressions, we then investigated the characteristics associated with these trajectories. Four distinct trajectories of caregiving were identified: “stable intensive”, “increasing intensive”, “decreasing”, and “stable no care”. Results suggest that although there are socioeconomic, demographic, and health differences across the trajectories of caregiving (with younger women in good health and poorer socioeconomic status more likely to care intensively throughout), family characteristics are their main drivers. Respondents who live alone, with no children, and no parents alive are more likely to never provide care, whereas those with older parents and who live with adults in poor health are more likely to provide stable intensive care. Also, changes in family characteristics (e.g. death of parents, widowhood, or deterioration of the partner’s health) are associated with trajectories representing increases or decreases in caregiving over time. Overall, trajectories of informal caregiving undertaken by older people are varied and these patterns are mostly associated with both the availability and health of family members, suggesting that need factors represent the most immediate reason for caregiving commitments.

**Supplementary Information:**

The online version contains supplementary material available at 10.1007/s10433-024-00818-w.

## Introduction

Informal caregivers provide unpaid care, often to family members or friends in need of support and care due to long-term physical disability, mental health conditions, or chronic disease. While the prevalence of informal caregiving depends on the definition used, recent estimates suggest that approximately 17% of the adult population in Europe provides informal care (Tur-Sinai et al. 2020), with those in late mid-adulthood (aged 50–64) often most likely to become informal caregivers and to make up the majority of caregivers (Lacey et al. 2024; Larkin et al. 2019; ONS 2023). Moreover, many caregivers provide several years of care; according to the UK Office for National Statistics, men and women aged 50 years can expect to spend 4.9 and 5.9 years, respectively, of their remaining life as unpaid caregivers (ONS 2017).

Although there is growing interest in caregiving research in later life and an increasing body of research exploits available longitudinal datasets, to date, most of these studies have focused on the consequences and sequelae of care provision (Evandrou et al. 2024; Larkin et al. 2019; Schulz et al. 2020). There is extensive evidence about the (generally negative) impact of care provision on caregivers’ mental and physical health, employment and finances, as well as social life (Bauer and Sousa-Poza 2015; Bom et al. 2018; Keating and Eales 2017; OECD 2011; Price and Di Gessa 2023). However, it is acknowledged that links between caregiving and caregiver outcomes vary by socioeconomic and demographic characteristics, the extent of and involvement in providing care, as well as the clinical conditions of and the relationship with the care recipients (Brown and Brown 2014; Zueras and Grundy 2024).

Longitudinal studies have often also investigated patterns of caregiving received (rather than *provided*) by older people and have shown how increases or reductions in the amount of care received by older people over time often occur in response to changes in people's functional status and the needs of individuals (Deeg et al. 2005; Miller and McFall 1991) as well as the availability and provision of publicly funded formal care (Kjær and Siren 2020; Li 2005). Although there is considerable heterogeneity in older people’s trajectories of frequency and intensity of care utilisation (Hu 2020), care receipt often increases over time after disease onset, particularly for those who suffer from degenerative conditions that require more assistance over time (Jutkowitz et al. 2020).

To date, however, few studies have analysed longitudinal patterns of caregiving (and the characteristics of those in different trajectories of caregiving) despite informal caregiving often being conceptualised as a dynamic process wherein several changes may occur (Schulz et al. 2020; Uccheddu et al. 2019; Verbakel and Glijn 2023). Indeed, depending on the care recipient’s functioning and needs and their relationship with the caregiver, care provision might involve different tasks and complexities of activities (from practical and emotional support to medical and self-care help), different frequencies and levels of intensity (from around-the-clock daily to sporadic one-off hours of care), and different progressions (that could increase, decrease, or even end over time depending on how critical and long-lasting the illness or need of the person cared for is.) Therefore, it is important to both describe the longitudinal trajectories of informal caregiving to better understand the dynamic nature of informal care in later life and to examine the characteristics and family situations of those experiencing different trajectories of caregiving.

To our knowledge, studies on the trajectories of informal caregiving have mostly used two time points to capture changes in informal care provision. For instance, drawing data from two longitudinal studies conducted in Massachusetts during the 1970s and 1980s, Jette and colleagues’ study (1992) was one of the first to examine stability and changes in caregiving patterns. Since then, several other studies have investigated these patterns of care but have mostly considered two time points and therefore distinguished between broad categories of “continuing”, “starting”, “stopping”, and “never” caregiving patterns (Lawton et al. 2000; Lee and Gramotnev 2007; McCann et al. 2004; Robards et al. 2015). Among the noticeable exceptions, Tooth and Mishra (2014) used data from the Australian Longitudinal Study on Women’s Health collected over 9–13 years (i.e. 4–5 waves of data) to identify trajectories of care provision. Among older cohort members, the authors identified three classes that distinguished between women with constantly low or high probabilities of being a caregiver and a third group of women who initially provided no care to then show a substantial increase in caregiving. The authors also identified individual demographic, socioeconomic, and health characteristics associated with these trajectories of caregiving, showing that women with relatively poorer socioeconomic backgrounds were more likely to provide continuing care throughout the 9-year period under study. Using retrospective data from a panel sample of Dutch caregivers aged 16–78, Verbakel and Glijn (2023) also showed heterogeneous trajectories of caregiving and identified three classes of care that represented a decrease, stability, and increase in care demands (assessed with care receivers’ health condition), intensity of care (i.e. number of informal caregiving hours and duration of care episodes), and care complexity (captured by number and types of caregiving tasks). The authors also found that different trajectories were related to both the age and the living arrangement of the care receiver.

According to the "Informal Care Model", becoming a caregiver (and subsequent provision of informal care) depends on the care receiver’s need for care; the individual predisposing and enabling factors (such as expectations around care, financial resources, and health); and external conditions (including availability of formal support) that can facilitate or restrict the provision of informal care (Broese van Groenou and De Boer 2016). Although the care recipient’s needs are assumed to be the most important drivers for the onset of and changes in informal caregiving, studies on changes in caregiving have thus far overlooked how and to what extent the trajectories of informal caregiving relate to the (changing) ability of the informal caregivers to provide care and happen in response to (changing) family situations and their potential needs of care.

In this paper, therefore, first, we describe, at a population level, patterns of informal caregiving over time among older people in England, moving beyond “snapshots” that are often used to describe care provision in later life and accounting for the intensity of care provided (Keating et al. 2019). Second, to examine the factors underpinning variations in care provision, we analyse which individual and family characteristics are associated with distinct trajectories of informal caregiving in later life. In particular, in this paper, we consider factors related not only to individuals’ likelihood of providing informal care (such as gender, financial resources, time constraints, or health (Baldassar et al. 2007; Bauer and Sousa-Poza 2015; Brouwer et al. 2005; Carmichael et al. 2010; Szinovacz and Davey 2008)) but also to care recipients’ needs (such as the presence and health of dependents who might require care). Taken together, our study aims to provide valuable additional insights into the dynamic nature of informal care provision and the impact of changing individual and need factors on caregiving over time.

## Methods

### Study design and participants

Data were obtained from the English Longitudinal Study of Ageing (ELSA). This is an ongoing multidisciplinary longitudinal nationally representative survey of individuals aged 50 years and older who live in private households in England (Banks et al. 2021). The specific details of the sampling frames and methodology, weighting strategies, and questionnaires can be found at www.elsa-project.ac.uk. ELSA started in 2002 and data are collected biennially via face-to-face personal interviews and self-completion questionnaires, with the most recent full wave of data collection occurring in 2018–19 (wave 9). Informed consent was obtained from all participants. All data are available through the UK Data Service (SN 5050).

Our sample consisted of non-proxy participants who had been successfully interviewed in both Wave 6 (2012/13) and Wave 9 (i.e. respondents who were still alive and had not dropped out of the study by 2018/9) and who had available information about care provision (the main variable of interest) in at least one wave. We did not include previous waves because the questions on informal care provision were asked differently and/or had different filters/routings. The final analytical sample consisted of 6561 ELSA participants (94% of whom were present in all four waves under study).

### Main measurements of interest

#### Outcome

In Waves 6–9 of ELSA, all respondents were asked two questions about the provision of care. In the first, respondents were asked (in the “Work and Pensions” module) if, among the activities performed in the previous month, they also “cared for someone”. They were then also asked whether they “looked after anyone in the past week”. Those who looked after someone in the week before the interview were then asked a series of follow-up questions including who they looked after, how many hours, how many people they cared for, and whether any of the care recipients lived with them. For the main variable of interest, respondents were then classified as “not providing care”, “providing intensive care” if they looked after someone they lived with or someone outside of the household for more than 10 h in the previous week, and “providing non-intensive care” if they cared for someone living outside of the household for fewer than 10 h in the previous week or if they did so in the previous month only.

#### Covariates

In line with the informal care model (Broese van Groenou and De Boer 2016), we accounted for a wide range of demographic, socioeconomic, health, and family covariates. All covariates were assessed at Wave 6, hereafter also referred to as “baseline” measurements. As demographic factors we considered gender and age modelled as a categorical variable, distinguishing those aged 50–59, 60–69, and 70 years and older. The socioeconomic factors included education, wealth, employment, and volunteering. Educational level was recoded into a binary variable distinguishing between low (below secondary) and middle/high education following the International Standard Classification of Education (http://www.uis.unesco.org/). Wealth was equal to the total net non-pension non-housing wealth, and respondents were categorised into wealth tertiles. For employment status, we classified respondents as being in paid work or not. Finally, respondents reported if they had volunteered in the month before the interview.

Health variables included self-perceived health, physical disability, depression, and multimorbidity. Self-rated health (SRH) was measured on a five-point ordinal scale (excellent, very good, good, fair, or poor). The five SRH items were dichotomised into “fair or poor” versus better health. Physical disability was assessed using limitations in activities of daily living (ADL, such as getting out of bed and walking across a room) and instrumental ADL (such as shopping for groceries and preparing a hot meal). Participants who reported limitations with one or more activities were defined as having a physical disability. For mental health, ELSA included an abbreviated eight-item version of the Centre for Epidemiologic Studies Depression Scale (CES-D) (Radloff 1977). Respondents are asked whether they had experienced any depressive symptoms, such as restless sleep or being unhappy, in the week before the interview, with those reporting four or more classified as "depressed" (Steffick 2000). Finally, we classified respondents as having multimorbidity if they reported two or more long-term medical conditions (including high blood pressure, coronary heart disease, stroke, diabetes, and cancer) (Zaninotto et al. 2020).

Among the indicators of family composition, we considered the presence of potential dependents who might require care as well as their health, where possible. In particular, we included indicators of whether respondents had any living siblings or not, whether they had any children, and among those with children whether at least one child lived with the respondent. For (biological) parents, ELSA does not collect information on their health; therefore, considering parents’ age as a crude health proxy, we classified respondents as having no parents alive, parents younger than 85 years, or at least one parent aged 85 years or older. Moreover, ELSA collects information on all consenting adults aged 50 + and respondents’ partners (regardless of their age). Exploiting the study design and using both the health indicators described above and the household composition, we constructed a variable that not only accounted for whether the respondents lived with other respondents aged 50 years and older (mostly their partners) but also whether that person was in overall good health.

#### Changes over time

As the covariates mentioned above were assessed in all ELSA waves, we also considered most of the social relationships and health indicators in terms of changes over the 6-year follow-up period. Depending on the variables and their distributions, we created variables capturing changes over time or disruptive events. For instance, for health-related variables, we considered categories such as “no change”, “health has improved”, and “health has deteriorated”, whereas for social indicators we constructed binary indicators capturing widowhood or the death of parent(s).

#### Statistical analysis

First, the percentages of respondents who provided care were calculated for each wave under study. In order to identify distinctive trajectory patterns of informal care provision, we used group-based trajectory modelling (Nagin 1999; Nagin and Odgers 2010); this method is used to cluster individuals into meaningful subgroups, each with a similar underlying trajectory of caregiving. This method takes into account the dependency of observations and assumes a mixture of subpopulations with different individual trajectories within the target population and identifies distinctive groups within which individuals share similar developmental trajectories (Herle et al. 2020; Nguena Nguefack et al. 2020). To determine the number of trajectory groups within our sample, we fit a series of group-based trajectory models with up to six groups. Missing data were handled using full information maximum likelihood estimation. When selecting the appropriate number of trajectory groups, we considered a wide range of criteria including the Akaike Information Criterion (AIC) and the Bayesian Information Criterion (BIC). For each of these, lower scores indicate (relatively) better fitting models. Moreover, we additionally considered the average posterior probabilities of group membership as a measure of classification quality; group size (and the avoidance of too small classes that may lead to a lack of reproducibility of the results); the usefulness of the number of groups in terms of the similarities/differences in their trajectories; and the interpretability of the distinctive trajectories (Nagin and Odgers 2010; Nguena Nguefack et al. 2020).

Once the trajectories were identified, we first examined the (unadjusted) differences among these trajectory groups in terms of demographic; socioeconomic; family; and health-related covariates at baseline (using Chi-squared tests). Second, we used multinomial logistic regression analyses to examine the combined effects of these characteristics on respondents’ group membership of different trajectories of informal caregiving. Third, we examined the associations between the trajectories of caregiving and changes in selected social relationships and health indicators (controlling for baseline basic sociodemographic characteristics). To ease the interpretation of the results, the findings are reported as average marginal effects (AMEs) for the explanatory variable. Due to the categorical nature of our outcomes and explanatory variables, the AMEs are interpreted as the discrete effect of the independent variable (compared to the reference category), i.e. as the difference between the predicted probabilities (in percentage points) across the groups being compared. Trajectories were determined using Mplus; data management and statistical analyses were performed using Stata 18.

## Results

### Distinctive trajectories of informal caregiving

Table 1 shows the distribution of the care provision categories across all four waves under study. Overall, each of the three categories of care provision calculated for ELSA participants shows stable probabilities over time. The majority of respondents (approximately three in four) were classified as not providing any care across all waves, with about 11/12% of respondents reporting intensive care at each wave.

**Table 1:** Per cent distribution of care provision at each wave under study. *Source*: English Longitudinal Study of Ageing (ELSA) Waves 6 (2012/13) – 9 (2018/19)

	N	No care	Non-intensive care	Intensive care
Wave 6	6547	4931 (75.3%)	783 (12.0%)	833 (12.7%)
Wave 7	6300	4805 (76.3%)	797 (12.6%)	698 (11.1%)
Wave 8	6221	4773 (76.7%)	697 (11.2%)	751 (12.1%)
Wave 9	6336	4901 (77.4%)	707 (11.2%)	728 (11.4%)

**Fig. 1:**
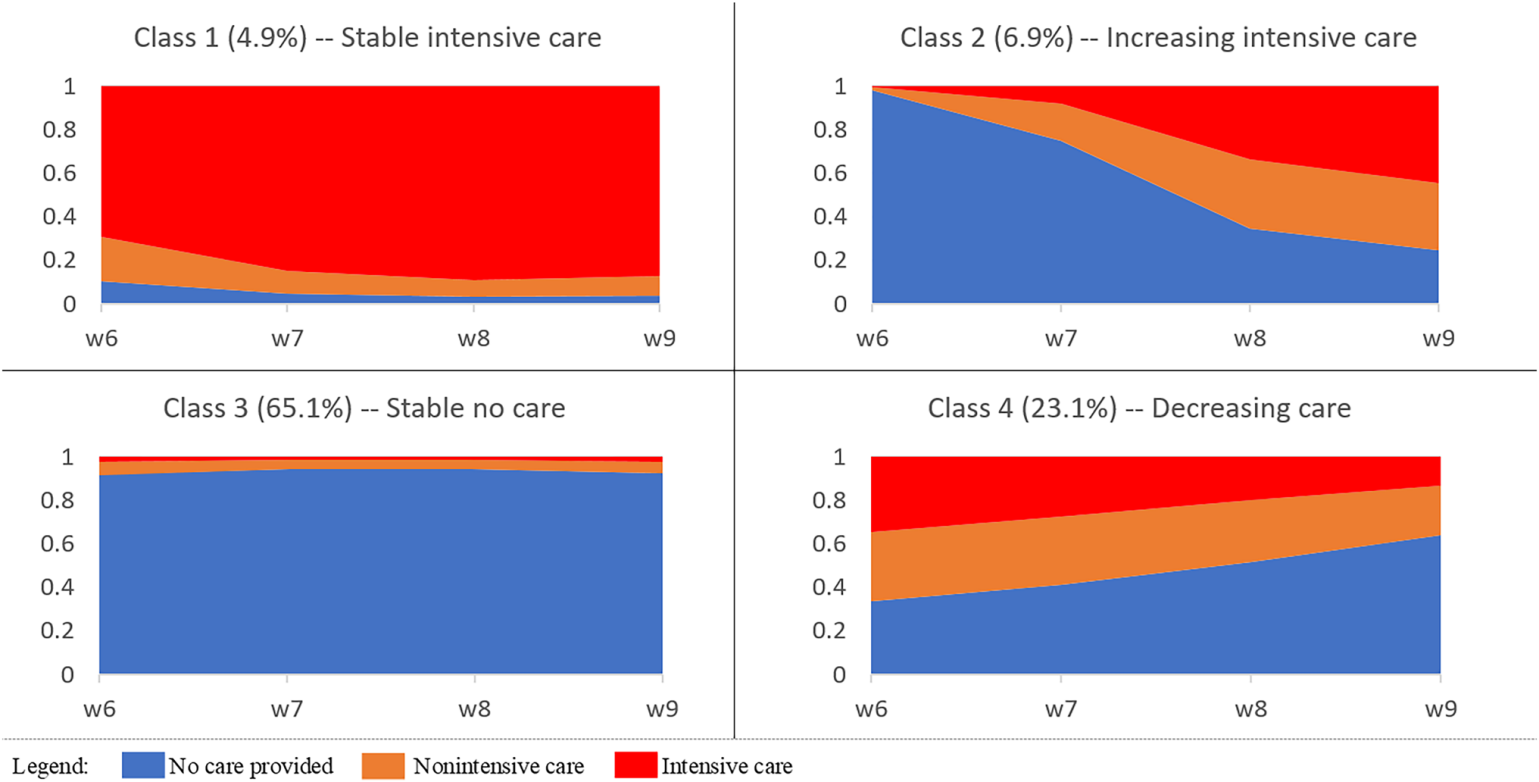
Stacked predicted probabilities of informal care provision (“No care”; “Non-intensive care”; and “Intensive care”). *N﻿otes*: These probabilities are predicted by the best-fitting group-based trajectory model with 4 classes. *Source* English Longitudinal Study of Ageing (ELSA) Waves 6 (2012/13) – 9 (2018/19). N = 6561

To summarise the dynamic process of informal care provision over time and determine the optimal number of trajectory groups, a series of group-based trajectory models were fitted (with a specification of up to six trajectory groups). Based on the goodness-of-fit criteria (shown in Supplementary Table 1) and the other considerations mentioned above, we identified four as the number of trajectories that best fit the data. The cumulative predicted probabilities of each of the three categories varied substantially across classes (Fig. 1). Class 1 (4.9%) and Class 3 (65.1%) identified groups of respondents who reported “intensive informal care” and “no informal care”, respectively, with high and fairly stable probabilities. The respondents in these groups are hereafter classified as “*stable intensive care*” and “*stable no care*”. The other two classes show time-varying probabilities: Class 2 (6.9%) comprises older people who initially do not provide informal care but who progressively look after people and do so more intensively over time (“*increasing intensive care*”), while Class 4 (23.1%) includes those whose probabilities of being informal caregivers (roughly equally split between intensive and non-intensive) steadily decrease over time (“*decreasing care*”).

Table 2 shows the characteristics of the care provided in the week before the interview at each wave for respondents in each of the different trajectories, excluding those who were clustered in the “*stable no care*” group. Overall, as one would expect, respondents in “*stable intensive care*” reported high average hours of care per week, with about one third providing care 24/7. Approximately three in four of those classified as “stable intensive caregivers” also lived with the person they care for who was, in the majority of the cases, their partner/spouse. Across the waves under study, the intensity of care provided by this group increased, and these respondents became more likely to care for just their partners. Those in the “*decreasing care*” group, on the other hand, experienced a reduction of their overall commitment to this task; these informal caregivers were most likely to care for friends and “other” family members and over time a smaller percentage cared for their parents/parents-in-law. Finally, after relatively modest initial engagement in this activity, “*increasing intensive care”* providers spent increasingly more time looking after people and were more likely to mention that they cared for someone they lived with, their spouse, or their parent(s)/in-law.

**Table 2:** Caregiving characteristics by trajectories of informal caregiving and waves.

	Wave 6	Wave 7	Wave 8	Wave 9
*Mean number of hours of care provided*				
Stable intensive care	67.5	66.6	71.9	81.8
Increasing intensive care	–	5.1	37.3	45.0
Decreasing care	32.6	32.8	28.6	26.0
*% Caregiving 24 h a week*				
Stable intensive care	32.6	32.0	34.8	40.8
Increasing intensive care	–	2.0	15.5	20.0
Decreasing care	12.6	12.9	10.6	9.5
*% Living with the person they cared for*				
Stable intensive care	72.7	74.1	79.6	80.5
Increasing intensive care	–	2.9	46.4	48.4
Decreasing care	33.5	33.3	31.1	25.3
*% Caregiving for 2 or more people*				
Stable intensive care	29.1	25.1	20.8	19.1
Increasing intensive care	–	37.1	28.3	26.9
Decreasing care	32.2	28.0	26.3	30.2
*% Caregiving for spouse/ partner*				
Stable intensive care	56.4	59.9	63.3	64.1
Increasing intensive care	–	2.9	39.2	42.3
Decreasing care	26.2	26.4	25.3	23.4
*% Caregiving for parents/ in-laws*				
Stable intensive care	19.4	17.8	15.5	12.7
Increasing intensive care	–	14.1	17.9	20.5
Decreasing care	24.6	21.3	17.7	17.1
*% Caregiving for a child*				
Stable intensive care	14.5	12.6	11.7	13.9
Increasing intensive care	–	0.0	4.6	4.6
Decreasing care	7.2	5.4	7.6	6.5
*Caregiving for other family/ friends*				
Stable intensive care	24.5	23.1	18.7	18.4
Increasing intensive care	–	30.0	30.4	30.7
Decreasing care	39.0	35.2	34.4	36.2

Caregiving characteristics refer to those who provided care for someone in the week before the interview and are not reported for respondents whose group-based trajectory class presented a relatively high probability of not providing care throughout the waves under study (“*stable no care*”). Percentages do not necessarily add to 100 as some respondents cared for two or more people. Values at Wave 6 for the “*increasing intensive care*” groups are not reported (–) because they are based on less than 30 respondents

*Source*: English Longitudinal Study of Ageing (ELSA) Waves 6 (2012/13) – 9 (2018/19). N = 6561

### Determinants of the trajectories of informal caregiving

Table 3 summarises ELSA respondents’ baseline characteristics and shows the distributions of their demographic, socioeconomic, health, and family characteristics among the four caregiving trajectory groups. Overall, men and people aged 70 yers and older were more likely to be in the “stable no care group”. Generally, those who cared intensively over time had the lowest levels of wealth and engagement in both paid and voluntary work. When health is considered, those in the “stable intensive care” group were more likely to report poor health whereas respondents in the “increasing intensive care” group (who were not initially engaged in caregiving activities) had the best health profile at baseline. Finally, as expected, respondents in the “stable no care” group were more likely to have no children, no parents alive, and to live on their own. Among those who lived with other people, “stable intensive” caregivers were overwhelmingly likely to reside with adults (mostly their partners) in poor health.

**Table 3:** Demographic, socioeconomic, health, and family characteristics of the ELSA sample by caregiving trajectories.

	Stable intensive care	Increasing intensive care	Stable no care	Decreasing care	Total	*P* value
Female	63.4	60.6	53.1	66.0	56.3	< 0.001
50–59	31.0	33.3	25.6	31.1	27.4	< 0.001
60–69	45.4	42.4	41.4	46.5	42.6	
70 +	23.6	24.2	33.0	21.5	30.1	
Medium/High Education	62.1	63.6	62.6	65.9	63.3	0.189
Top wealth tertile	22.8	39.2	37.5	39.0	37.2	< 0.001
Middle wealth tertile	38.4	38.1	33.0	32.2	33.1	
Lowest wealth tertile	38.8	22.7	29.5	28.8	29.5	
In work	26.8	40.5	35.2	35.9	35.1	< 0.001
Voluntary work	15.9	20.5	16.5	23.9	18.0	< 0.001
Fair/poor SRH	25.7	14.8	23.7	17.7	22.3	< 0.001
Depressed	13.4	10.6	11.4	11.4	11.5	0.744
Disability	28.9	18.9	21.1	16.3	20.4	< 0.001
Multimorbidity	22.9	11.0	17.2	14.4	16.7	< 0.001
No children	8.8	9.1	13.7	8.5	12.3	< 0.001
Children out HH	77.1	75.0	76.1	80.2	76.8	
1 + Child in HH	14.1	15.9	10.3	11.4	11.4	
Has brothers/sisters alive	77.5	84.1	81.2	81.9	81.3	0.217
No parents alive	70.4	65.5	80.0	64.6	76.1	< 0.001
Parent(s) aged < 85	11.6	17.8	9.9	16.0	11.5	
1 + parent aged 85 +	18.0	16.7	10.1	19.4	12.4	
Living alone	3.4	10.5	25.4	14.3	21.9	< 0.001
Living with other/info missing	9.0	9.0	8.6	9.8	8.8	
Other in Fair/Poor Health *	60.4	31.0	21.6	32.8	26.4	< 0.001
Other Depressed *	26.6	9.0	6.8	10.3	8.7	< 0.001
Other Disabled *	57.6	24.1	13.9	27.5	19.5	< 0.001
Other with Multimorbidity *	29.9	16.6	13.0	16.3	14.7	< 0.001
Total Respondents—N	284	264	4797	1216	6561	

*These percentages are restricted to respondents living with another adult with available information

*P*-value were calculated from Chi-squared tests

*Source*: English Longitudinal Study of Ageing (ELSA) Waves 6 (2012/13) – 9 (2018/19)

Table 4 shows the results from the multinomial logistic regression analyses regarding demographic, socioeconomic, health, and family factors measured at baseline as predictors of the trajectories of informal care. The results suggest that women were more likely than men to be in the “stable intensive” (1.3 percentage points) or “decreasing” (7.6 percentage points) care trajectories, whereas they were 9.8 percentage points less likely to be in the “stable no care” group, after adjustment for other explanatory variables. People aged 70 years and older at baseline were more likely to be in the “no care” trajectory and less likely to be in the “decreasing” or “stable intensive” caring trajectories than respondents in their 50 s. There were also some socioeconomic differences across the trajectories of informal caregiving: for instance, those in the top wealth tertile distribution were more likely to be in the “stable no care” group and less likely to be in the “stable intensive care” group. Similarly, compared to those not in paid work, respondents in paid work were about 7 percentage points more likely to be in the “stable no care” group, but around 3 percentage points less likely to provide stable intensive care. Engagement in voluntary work was also related to trajectories of caregiving, with those who volunteered in the month before the interview being 8.8 percentage points less likely to be in the “stable no care” group and 7.9 percentage points more likely to be in the “decreasing” care group. Those who rated their health as poor at baseline were more likely to be in the “stable no care” group and less likely to be in any of the three remaining trajectories of caregiving. Finally, as the descriptive statistics suggested, family composition and health were strongly related to the trajectories of caregiving. People with children and parents alive were up to about 7 and 16 percentage points less likely, respectively, to be classified in the group “stable no care” compared to those without children and parents. However, respondents with siblings were more likely to be classified as “stable no care” and less likely to be classified as “stable intensive” caregivers. Respondents with parents alive were up to 12.5 percentage points more likely to be in the group whose caregiving commitment declines over time. Similarly, living with other people was generally associated with higher probabilities of being in one of the three trajectories of caregiving but the health profile of the adult respondents lived with matters. For instance, if the adult they resided with was in poor self-rated health, respondents were 11.1 percentage points more likely to be in the “stable intensive” care category and 13.2 percentage points more likely to be in the “decreasing care” group (suggesting that at baseline, living with someone with poor health increased dramatically the likelihood of providing care). However, if the health of the adult they lived with was rated as good, the percentage points to be in these two groups were much smaller (2.5 and 5.3, respectively). Very similar results and patterns were also observed when the co-residing adults’ disability, depression, and multimorbidity statuses were considered (see Supplementary Table 2 for details).

**Table 4:** Fully adjusted average marginal effects (with 95% CIs) for the relationships between demographic, socioeconomic, health, and family characteristics and trajectories of informal caregiving.

	Stable intensive care	Increasing intensive care	Stable no care	Decreasing care
Female^a^	1.26* [0.28; 2.24]	0.91 [− 0.07; 1.89]	− 9.77*** [− 11.9; − 7.66]	7.59*** [5.72; 9.46]
60–69^b^	− 1.04 [− 2.69; 0.60]	0.11 [− 1.31; 1.53]	1.19 [− 2.05; 4.44]	− 0.26 [− 3.18; 2.66]
70 + ^b^	− 2.50** [− 4.28; − 0.72]	− 0.02 [− 1.80; 1.76]	8.28*** [4.50; 12.1]	− 5.81*** [− 9.16; − 2.48]
Medium/High education^c^	0.35 [− 0.67; 1.38]	− 0.47 [− 1.55; 0.60]	− 0.53 [− 2.78; 1.72]	0.65 [− 1.36; 2.66]
Mid wealth tertile^d^	− 0.57 [− 1.99; 0.85]	1.35* [0.09; 2.60]	0.37 [− 2.41; 3.16]	− 1.15 [− 3.65; 1.34]
Top wealth tertile^d^	− 2.92*** [− 4.24; − 1.60]	0.74 [− 0.50; 1.98]	3.30* [0.46; 6.14]	− 1.11 [− 3.69; 1.45]
In paid work^e^	− 2.70*** [− 3.83; − 1.57]	0.04 [− 1.26; 1.18]	7.25*** [4.71; 9.78]	− 4.51*** [− 6.76; − 2.25]
Voluntary work^f^	0.27 [− 1.12; 1.66]	0.64 [− 0.69; 1.99]	− 8.78*** [− 11.7; − 5.87]	7.86*** [5.16; 10.6]
Depressed^g^	− 0.88 [− 2.24; 0.48]	0.40 [− 1.36; 2.16]	− 0.96 [− 4.51; 2.58]	1.44 [− 1.78; 4.67]
Fair/poor SRH^h^	− 1.25* [− 2.42; − 0.08]	− 1.57* [− 2.76; − 0.38]	6.68*** [3.91; 9.44]	− 3.86** [− 6.35; − 1.36]
Disability^i^	1.64* [0.18; 3.10]	0.99 [− 0.58; 2.56]	0.37 [− 2.56; 3.30]	− 3.00* [− 5.52; − 0.47]
Multimorbidity^j^	1.41 [− 0.07; 2.89]	− 1.00 [− 2.32; 0.32]	− 0.64 [− 3.69; 2.40]	0.23 [− 2.54; 3.01]
Child(ren) live out HH^k^	2.13 [− 1.43; 1.86]	0.56 [− 0.92; 2.04]	− 6.82*** [− 9.97; − 3.65]	6.04*** [3.34; 8.73]
1 + Child(ren) in HH^k^	1.35 [− 0.88; 3.59]	1.54 [− 0.54; 3.62]	− 6.01** [− 10.3; − 1.73]	3.12 [− 0.50; 6.73]
Has brothers/sisters^l^	− 2.04** [− 3.56; − 0.52]	0.36 [− 0.90; 1.62]	3.15* [0.33; 5.95]	− 1.46 [− 4.00; 1.07]
Parent(s) < 85^ m^	0.34 [− 1.39; 2.07]	2.14* [0.05; 4.23]	− 11.7*** [− 15.9; − 7.59]	9.27*** [5.43; 13.1]
Parent(s) > = 85^ m^	2.39* [0.55; 4.24]	1.52 [− 0.16; 3.19]	− 16.4*** [− 20.0; − 12.8]	12.5*** [9.15; 15.9]
With adult: good SRH^n^	2.54*** [1.78; 3.29]	2.08*** [0.97; 3.20]	− 9.90*** [− 12.5; − 7.31]	5.27*** [2.91; 7.63]
With adult: poor SRH^n^	11.1*** [9.32; 13.0]	3.60*** [2.01; 5.18]	− 27.9*** [− 31.3; − 24.5]	13.2*** [10.1; 16.2]
With adult (missing)^n^	3.28*** [1.61; 4.95]	1.54 [− 0.35; 3.43]	− 10.6*** [− 15.0; − 6.28]	5.81** [1.91; 9.72]

*N* = 6424. The values in brackets are the 95% Confidence Intervals (CIs). *p < 0.05; **p < 0.01; ***p < 0.001

*HH* household; *SRH* Self-Rated Health. Reference categories are ^a^Male; ^b^50-59 years; ^c^Low education; ^d^Low wealth tertile; ^e^Not in paid work; ^f^No voluntary work; ^g^Not depressed; ^h^At least good SRH; ^i^No disability; ^j^No multimorbidity; ^k^No children; ^l^No brothers or sisters alive; ^m^No parents alive; ^n^Living alone

*Source*: English Longitudinal Study of Ageing (ELSA) Waves 6 (2012/13) – 9 (2018/19)

Finally, Table 5 shows the results from the multinomial logistic regression analyses regarding the associations between changes in selected family and health compositions and the trajectories of informal caregiving. Once again, the findings are reported as AMEs; the per cent distributions of each variable are available in Supplementary Table 3. Overall, there was little evidence of an association between changes in personal health and those trajectories of caregiving that also showed changes in the predicted probability of providing informal care over time. However, the results suggest that those who had become depressed over the years under study were more likely to be classified in the “stable intensive care” group (and less in the “stable no care” group) compared to those who experienced no changes in their depressive symptomatology. When family is considered, we found significant associations between changes in family characteristics and changes in caregiving over time. For instance, respondents who lost their parents during the years under study were 12 percentage points more likely to be classified in the “decreasing care” group compared to those without parents or whose parents were still alive. Similarly, respondents whose household composition changed and who lived alone by wave 9 (and who had mostly become widowed) were 3 percentage points less likely to be in the “increasing intensive care” group but almost 15 percentage points more likely to be in the “decreasing care” group. Similarly, when changes to the health profile of the adults that respondents live with were taken into account, the results suggest, unsurprisingly, that the deterioration in the physical health of the co-residing adult was associated with higher percentages of belonging to the “increasing intensive care” group, whereas if the co-residing adult’s health improved respondents were more likely to experience a decreasing caregiving trajectory.

**Table 5:** Fully adjusted average marginal effects (with 95% CIs) for the relationship between changes in selected health and family characteristics between wave 6 and wave 9 and the trajectories of informal caregiving.

	Stable intensive care	Increasing intensive care	Stable no care	Decreasing care
No changes in depression	Ref			
Has become depressed	3.21** [0.74; 5.69]	0.02 [− 1.92; 1.97]	− 5.38* [− 9.90; − 0.86]	1.91 [− 2.01; 5.83]
No longer depressed	1.33 [− 0.87; 3.54]	0.61 [− 1.47; 2.71]	− 1.58 [− 5.99; 2.82]	0.37 [− 4.14; 3.40]
No changes in SRH	Ref			
Has become with poor SRH	0.93 [− 0.80; 2.67]	− 0.65 [− 2.14; 0.84]	− 2.77 [− 6.33; 0.77]	2.49 [− 0.69; 5.67]
No longer with poor SRH	− 0.07 [− 2.01; 1.86]	− 1.63 [− 3.39; 0.12]	3.53 [− 0.66; 7.73]	− 1.82 [− 5.53; 1.89]
No changes in disability	Ref			
Has become disabled	0.54 [− 0.97; 2.07]	− 1.19 [− 2.52; 0.14]	0.80 [− 2.34; 3.96]	− 0.06 [− 2.88; 2.75]
No longer disabled	3.27* [0.77; 5.78]	2.04 [− 0.34; 4.42]	− 7.62** [− 12.2; − 2.98]	2.30 [− 1.68; 6.29]
No changes in multimorbidity	Ref			
Has reported multimorbidity	0.19 [− 1.57; 1.96]	− 0.47 [− 2.07; 1.12]	0.68 [− 3.03; 4.39]	− 0.40 [− 3.69; 2.89]
No longer with multimorbidity	0.96 [− 2.09; 4.03]	0.21 [− 2.56; 2.99]	1.51 [− 4.42; 7.44]	− 2.69 [− 7.73; 2.34]
Parent(s) died	1.19 [− 0.41; 2.80]	0.16 [− 1.26; 1.60]	− 12.9*** [− 16.4; 9.4]	11.6*** [8.32; 14.7]
No co-resident health changes	Ref			
Lives alone at wave 9	− 3.04*** [− 4.27; − 1.81]	− 2.82*** [− 4.05; − 1.61]	− 9.48*** [− 14.1; − 4.87]	15.4*** [10.8; 19.8]
With adult: poor SRH at wave 9	2.43* [0.20; 4.66]	4.51*** [2.12; 6.90]	− 12.0*** [− 15.2; 6.77]	3.34 [− 1.09; 7.78]
With adult: no longer in poor SRH at wave9	− 0.11 [− 2.35; 2.11]	1.39 [− 1.05; 3.84]	− 4.61 [− 9.67; 0.43]	5.06** [1.41; 8.71]
With adult: disabled at wave 9	3.58** [1.46; 6.24]	6.71*** [4.06; 9.38]	− 12.2*** [− 16.4; − 7.92]	1.60 [− 1.95; 5.16]
With adult: no longer disabled at wave 9	− 1.00 [− 3.07; 1.07]	1.36 [− 1.12; 3.85]	− 6.02 []	5.66* [0.89; 10.4]
With adult: multimorbidity at w9	2.81* [0.21; 5.42]	3.23* [0.46; 6.01]	− 8.77*** [− 13.7; − 3.85]	2.72 [− 1.43; 6.89]
With adult: no longer multimorbidity at wave 9	1.38 [− 2.07; 4.83]	3.04 [− 0.81; 6.98]	− 5.35 [− 12.2; 14.6]	1.35 [− 5.47; 5.74]

Changes are obtained by comparing the characteristics of waves 6 and 9. Those who “became unhealthy” were respondents who reported a health condition at wave 9 but not at wave 6. On the other hand, those who reported health conditions at wave 6 but not at wave 9 were classified as “no longer unhealthy”. The same principle applies to changes in household composition and to the health of co-residing adults. All sets of multinomial logistic regressions adjusted for gender, age groups, education, and wealth at wave 6. The values in brackets are the 95% Confidence Intervals (CIs). *p < 0.05; **p < 0.01; ***p < 0.001

*Source*: English Longitudinal Study of Ageing (ELSA) Waves 6 (2012/13) – 9 (2018/19)

## Discussion

In the context of an ageing population, combined with long-standing challenges in the delivery of formal social care for older people, unpaid caregivers play a key role in promoting the quality of life of older people and their extended families and ensuring that needs for care and support are met. Although the provision of informal care is often a process, most studies have provided snapshots of caregiving, overlooking its dynamic nature. Using data spanning 6 years from the nationally representative ELSA, we aimed to describe trajectories of caregiving in later life and the factors associated with them.

Overall, we found four distinct trajectories of informal caregiving with two-thirds of the sample under study never engaging in care provision throughout the 6 years under study, 5% providing intensive care throughout, and the remaining 30% showing a decreasing (23%) or increasing (7%) trajectory of informal care provision. These results show heterogeneity and complexity in the provision of informal care in later life, as reported in studies conducted in Australia and The Netherlands (Tooth and Mishra 2014; Verbakel and Glijn 2023). However, the number and prevalence of trajectories of caregiving in those studies are slightly different—this might be influenced not only by the measures and operationalisations of informal care provisions in the study, but also by external conditions (including formal care provision, generational differences in attitudes towards informal care, and employment policies) that can facilitate or restrict the provision of care in later life (Albertini and Kohli 2013; Price et al. 2018; van Damme and Spijker 2024).

This study also investigated the associations between demographic, socioeconomic, health, and family indicators and the trajectories of caregiving. We found that some of the personal demographic and socioeconomic characteristics were related to caregiving trajectories during the 6 years considered. For instance, in line with previous studies suggesting that family caregiving remains a predominantly “feminine” activity because of the gendered nature of different tasks, expectations of behaviours, responsibilities, and social structures and norms (Di Gessa et al. 2020; Haberkern et al. 2015; Sharma et al. 2016), we also found that women were generally more likely to belong to a caregiving trajectory. Moreover, our results indicate that people with poorer socioeconomic status (in the lowest wealth tertile and not in paid work) were more likely to have provided informal care intensively throughout the observation window, as also found in Tooth and Mishra (2014). It has often been argued that people with fewer resources and time constraints might have less to “lose” by becoming caregivers compared to those in employment or better off who have the resources to access, purchase, and use alternative forms of care, help, and support from the market (de Zwart et al. 2017; Di Gessa et al. 2022a, b; Quashie et al. 2022; Saito et al. 2018). We also found that health factors were associated with trajectories of caregiving: those in good self-rated health at baseline were generally more likely to have provided care in the 6 years under study, with some indication that reporting disabilities was related to the “stable intensive” care group. These baseline differential health associations could reflect both the selection into and consequences of these trajectories of informal caregiving as well as depend on the type of caregiving performed (Price and Di Gessa 2023; Wolff et al. 2016; Zwar et al. 2018).Overall, changes in personal health were not associated with changing trajectories of care provision. However, those who became depressed during the waves under study were more likely to belong to the “stable intensive care” group suggesting that changes in depression are a consequence of substained intensive care provision rather than a determinant of caregiving, as reported by other studies that have investigated the detrimental mental health consequences of caregiving in later life (Bom et al. 2018; Hiel et al. 2015). Finally, it is worth mentioning the characteristics of respondents in the “stable no care” group who were more likely to be older, in poorer self-reported health, to live alone, not to have parents and children but more likely to have siblings as well as to be in the highest wealth tertile and in paid work. For this group, lack of care provision might be partly driven by the fact that they lack people in need of care, partly by the fact that “others” might be providing that care (either formally, as this group of respondents has the means to buy care from the market, or informally by their siblings).

Not surprisingly, however, our results show that the need for care—operationalised in this study with the availability and health of family members—was the most dominant factor associated with trajectories of caregiving. This is in line with all models that position family care provision as stemming from having a close kin or friend who needs care (Brandt and Deindl 2017; Broese van Groenou and De Boer 2016). We found that older people who lived alone, had no children, and had no parents alive were more likely not to have provided any informal care during the ELSA waves examined, whereas those with older parents and those who lived with adults in poor health were more likely to report providing stable intensive care throughout the six years examined. Additionally, our results suggest that changes in the trajectories of caregiving were mostly related to changes in family circumstances. For instance, older people who experienced losses of parents and/or partners during the observation window of this study were most likely to belong to the “decreasing” caregiving trajectory, whereas those whose partner’s health deteriorated over time were significantly associated with the “increasing” trajectory of informal care provision.

### Strengths and limitations

We described the trajectories of caregiving by older English people over a period of 6 years, and the sociodemographic, health, and family factors associated with these trajectories. Although the availability of parents and spouses as well as their health progression (and therefore of their needs and demands) are the main theoretical drivers of onset and changes in informal care provision in later life (Brandt et al. 2009; Broese van Groenou and De Boer 2016), studies on changes in caregiving in later life have overlooked this aspect and have mostly focused on caregivers’ sociodemographic, economic, and health factors. To our knowledge, this was the first study to investigate this issue using a large scale nationally representative prospective survey that also accounted for a wide range of family characteristics, including the presence of parents and children, and the health of cohabiting adults. Our study demonstrated that care provision evolves over time, highlighting the limitations of a knowledge base founded on single care episodes, and that need factors are most likely to relate to trajectories of informal caregiving.

Our analyses, however, also have some limitations. ELSA does not collect detailed information about the care provided to each recipient but rather asks generic questions (related to all recipients of care) and the time spent looking after them. Therefore, in our trajectories and particularly for those who care for more than one person, we could not distinguish between different intensities of care or focus on specific care recipients. Moreover, we lack detailed information on the recipient of care: except for (the majority of) those who look after their cohabiting spouse/partner, we do not know for instance where the recipients of care live or their health status. Additionally, the caregiver-care recipient relationship, information on whether anyone else is involved in the provision of care (including other family members or friends as well as formal care providers), personal preferences for informal care (provision and receipts), and personality factors are all missing and would be useful for better describing and distinguishing trajectories of caregiving. More generally, ELSA lacks information on the broad domains and multiple tasks and activities that characterise family caregiving (ranging from assistance with daily activities and providing direct care to the recipient to navigating complex health care and providing emotional or practical help with paperwork). Although some information on the intensity of care is provided, most information refers to care provided in the week prior to the interview with little understanding of whether this was a one-off or more regular commitment. Similarly, 2-yearly surveys might miss more sporadic caregiving trajectories. Furthermore, we restricted our sample to those who were alive for the whole period under study; as those providing (intensive) care and those who lost a partner are more likely to have dropped out of the study, we might have overestimated the percentage of older people in the “no informal care” group. Moreover, although other studies in The Netherlands and Australia have shown similar patterns of caregiving (Tooth and Mishra 2014; Verbakel and Glijn 2023), we acknowledge that the trajectories found in our study and the factors associated with them may vary across countries with different formal long-term care settings and availability of formal support via the community. Furthermore, in our study we did not control for family-norms at either the individual or country level, including gender-related expectations about care, attitudes towards the norm that family should be responsible for care, the extent to which care is provided out of affection, altruistic behaviour, and reciprocity, or the degree to which someone feels ‘obliged’ or ‘expected’ to provide care from societal, cultural, or family pressures (Al-Janabi et al. 2018; Greenwood and Smith 2019). Given that informal care provision can also be time-specific and influenced by external factors (as the recent COVID-19 pandemic has shown (Chatzi et al. 2020; Di Gessa et al. 2022a, b; Price and Di Gessa 2023)), it is worth mentioning that our analyses are based on a snapshot of a specific timeframe (covering 6 years) of the respondents’ lifecourses, with baseline family circumstances, health, and ages being very heterogeneous across respondents. Moving forward, studies should also assess whether trajectories of caregiving differ across different cohorts and to what extent they relate to long-term health trajectories, as both issues were beyond the scope of this study.

## Conclusion

To conclude, our study shows that providing informal care in later life is a dynamic process, with one in 20 older people providing intensive care throughout a period of 6 years and 30% changing their probability of providing informal care, with both increasing and decreasing caregiving experience over time. Although personal sociodemographic and health characteristics are useful factors associated with the trajectories of informal caregiving, our results suggest that the availability of family (and potentially friends) and their needs and health profiles are the main drivers that shape the trajectories of informal care in later life. However, future research should aim to further investigate whether and to what extent trajectories of caregiving could be qualitatively distinct depending on the recipients’ specific health characteristics, the relationship with the recipient, and, more broadly, arrangements with other family members or friends or other commitments including grandchild care provision or employment. Furthermore, it would be interesting to investigate how the trajectories of informal care relate to the availability and use of formal care.

### Supplementary Information

Below is the link to the electronic supplementary material.

Supplementary file1 (DOCX 24 KB)

## Data Availability

Researchers can download all waves of ELSA data from the UK Data Service (SN: 5050, http://doi.org/10.5255/UKDA-SN-5050-27). For more information on how to access ELSA visit https://www.elsa-project.ac.uk/accessing-elsa-data

## Code availability

All analyses were performed using Mplus and Stata.
