# Supplementary material for: Determinants of trajectories of informal caregiving in later life: evidence from England
Source: Eur J Ageing. 2024 Aug 31;21(1):24. doi: 10.1007/s10433-024-00818-w (PMC11365911; doi:10.1007/s10433-024-00818-w)
Supplement: Supplementary file 1 — Supplementary file1 (DOCX 24 KB) [file 10433_2024_818_MOESM1_ESM.docx]

**Supplementary Table 1. Comparison of the goodness of fit criteria for group-based trajectory models of longitudinal informal care provision**

| **N classes** | **AIC** | **BIC** | **cBIC** | **Entropy** | **Class membership** |
| --- | --- | --- | --- | --- | --- |
| 1 | 36069.43 | 36096.59 | 36083.88 | 1 | 100.0% |
| 2 | 33116.47 | 33170.78 | 33145.36 | 0.733 | 24.7%; 75.3% |
| 3 | 32772.27 | 32853.73 | 32815.61 | 0.708 | 30.8%; 4.8%; 64.4% |
| **4** | **32643.00** | **32751.62** | **32700.78** | **0.785** | **4.9%; 6.9%; 65.1%; 23.1%** |
| 5 | 32593.69 | 32729.47 | 32665.92 | 0.680 | 2.1%, 52.0%, 26.4%, 16.3%, 3.2% |
| 6 | 32554.94 | 32717.88 | 32641.62 | 0.701 | 1.6%, 52.9%, 26.6%, 1.9%, 3.0%, 14.0% |

Source: English Longitudinal Study of Ageing (ELSA) Waves 6 (2012/13) – 9 (2018/19).

Notes: AIC=Akaike Information Criterion; BIC=Bayesian Information Criterion; c-BIC=sample size corrected BIC. N=6,561

**Supplementary Table 2. Fully adjusted average marginal effects (with 95% CIs) for the relationship between three different coresident health characteristics and trajectories of informal caregiving**

|  | ***Stable intensive care*** | ***Increasing intensive care*** | ***Stable no care*** | ***Decreasing care*** |
| --- | --- | --- | --- | --- |
| With adult: no disability | 2.33***  [1.60; 3.05] | 2.06***  [0.96; 3.17] | -8.90***  [-11.5; -6.34] | 4.51***  [2.18; 6.85] |
| With adult: disability | 14.7***  [12.3; 17.1] | 3.96***  [2.13; 5.79] | -36.6***  [-40.3; -32.8] | 17.9***  [14.4; 21.5] |
|  |  |  |  |  |
| With adult: no depressed | 4.16***  [3.34; 4.98] | 2.45***  [1.36; 3.54] | -13.4***  [-15.9; -10.8] | 6.75***  [4.46; 9.04] |
| With adult: depressed | 15.8***  [11.9; 19.7] | 2.74*  [0.36; 5.12] | -31.0***  [-36.4; -25.6] | 12.4***  [7.56; 17.3] |
|  |  |  |  |  |
| With adult: no multimorbidity | 4.24***  [3.40; 5.09] | 2.42***  [1.32; 3.52] | -13.1***  [-15.6; -10.5] | 6.40***  [4.08; 8.73] |
| With adult: multimorbidity | 9.09***  [7.02; 11.2] | 2.91**  [1.11; 4.71] | -24.7***  [-28.7; -20.8] | 12.7***  [9.14; 16.3] |

English Longitudinal Study of Ageing (ELSA) Waves 6 (2012/13) – 9 (2018/19). Notes: Values in brackets show the 95% CIs. *P<0.05; **p<0.01; ***p<0.001. All three sets of analyses adjusted for gender, age groups, education, wealth, employment, volunteering, depression, self-rated health, disability, multimorbidity, and the availability of children and parents.

**Supplementary Table 3. Changes in selected characteristics between wave 6 and wave 9 by caregiving trajectories**

|  | ***Stable intensive care*** | ***Increasing intensive care*** | ***Stable no care*** | ***Decreasing care*** | ***Total*** | ***P value*** |
| --- | --- | --- | --- | --- | --- | --- |
| No changes in depression | 79.8 | 85.5 | 87.3 | 86.8 | *86.6* | 0.039 |
| Has become depressed | 11.9 | 6.9 | 6.5 | 7.5 | *6.9* |  |
| No longer depressed | 8.3 | 7.6 | 6.2 | 6.7 | *6.5* |  |
| No changes in SRH | 79.8 | 85.9 | 81.4 | 82.0 | *81.6* | 0.230 |
| Has become with poor SRH | 13.0 | 9.9 | 11.4 | 12.2 | *11.6* |  |
| No longer with poor SRH | 7.2 | 4.2 | 7.2 | 5.8 | *6.8* |  |
| No changes in disability | 75.0 | 81.5 | 79.6 | 80.3 | *79.6* | 0.002 |
| Has become disabled | 13.7 | 9.0 | 14.5 | 12.7 | *13.9* |  |
| No longer disabled | 11.3 | 9.5 | 5.9 | 7.0 | *6.5* |  |
| No changes in multimorbidity | 87.0 | 88.6 | 86.8 | 88.7 | *87.3* | 0.984 |
| Has reported multimorbidity | 9.2 | 7.9 | 9.7 | 8.6 | *9.4* |  |
| No longer with multimorbidity | 3.8 | 3.4 | 3.5 | 2.7 | *3.3* |  |
| Parent(s) died | 18.6 | 15.9 | 11.1 | 22.1 | *13.6* | <0.001 |
| No co-resident health changes | 79.6 | 74.2 | 82.0 | 72.8 | *79.9* | <0.001 |
| Lives alone at wave 9 | 2.5 | 2.3 | 6.0 | 11.8 | *6.8* |  |
| With adult: no longer in poor SRH at wave 9 | 4.9 | 6.4 | 4.8 | 5.6 | *5.0* |  |
| With adult: poor SRH at wave 9 | 13.0 | 17.1 | 7.2 | 9.8 | *8.3* |  |
| With adult: no longer disabled at wave 9 | 3.5 | 6.1 | 4.7 | 5.8 | *4.9* | <0.001 |
| With adult: disabled at wave 9 | 14.8 | 20.1 | 7.2 | 8.5 | *8.3* |  |
| With adult: no longer multimorbidity at w9 | 3.5 | 4.7 | 2.8 | 2.7 | *2.9* | <0.001 |
| With adult: multimorbidity at wave 9 | 9.5 | 9.5 | 5.2 | 6.2 | *5.7* |  |

Source: English Longitudinal Study of Ageing (ELSA) Waves 6 (2012/13) – 9 (2018/19).

Notes: Changes are obtained by comparing characteristics at wave 6 and wave 9. Those who “became unhealthy” are respondents who reported the health condition at wave 9 but not at wave 6. On the other hand, those who reported health conditions at wave 6 but not at wave 9 are classified as “healthy”. The same principle applies to changes in household compositions and to the health of the co-residing adult. P values were obtained from multinomial logistic models that controlled for age, gender, education, and wealth at wave 6.
